# A Novel Lateral Tibiofibular Index for Simulated Posterior Fibular Translation: A Three-Dimensional CT Study

**DOI:** 10.3390/jcm15187262

**Published:** 2026-09-18

**Authors:** Oğuzhan Tanoğlu, Hamit Çağlayan Kahraman

**Affiliations:** 1 Department of Orthopaedics and Traumatology, Izmir Democracy University, 35140 Izmir, Türkiye; 2 Department of Orthopaedics and Traumatology, Fatih Sultan Mehmet Training and Research Hospital, University of Health Sciences, 34752 Istanbul, Türkiye; drhcaglayan@gmail.com

**Keywords:** ankle imaging, computed tomography, fibular translation, radiographic measurement, syndesmosis

## Abstract

**Background/Objectives**: The diagnosis of syndesmotic instability remains challenging, particularly in quantifying posterior fibular translation. This study aimed to assess the Lateral Tibiofibular Index (LTI), a novel lateral projection–based parameter, using standardized projections derived from three-dimensional computed tomography (3D CT) models. **Methods**: This retrospective simulation study included 80 adults (160 ankles; 40 women and 40 men; mean age, 42.7 ± 12.4 years). Native 3D models were used to generate standardized lateral projections, after which isolated posterior fibular translations of 1 mm and 2 mm were simulated. Three linear parameters (A, B, C) were defined based on fixed anatomical landmarks. The LTI was calculated using the formula (A + B)/(B + C). Four ratio-based candidate indices were evaluated. Receiver operating characteristic (ROC) analyses used participant-level clustered resampling to account for bilateral and repeated observations. **Results**: Mean LTI increased from 1.306 ± 0.099 in native ankles to 1.406 ± 0.109 after 1 mm translation and 1.515 ± 0.121 after 2 mm translation. The LTI showed an area under the curve (AUC) of 0.756 (95% confidence interval [CI], 0.727–0.793) for native versus 1 mm translation and 0.916 (95% CI, 0.885–0.946) for native versus 2 mm translation. Youden-derived thresholds were 1.357 (sensitivity, 67.5%; specificity, 74.4%) and 1.381 (sensitivity, 89.4%; specificity, 80.6%), respectively. **Conclusions**: The LTI showed promising simulation-based discrimination of native anatomy from controlled posterior fibular translation, particularly at 2 mm. An LTI of approximately 1.50 should be regarded as a provisional upper reference value rather than a validated diagnostic cut-off. Prospective clinical studies in patients with confirmed syndesmotic injury are needed to further evaluate the diagnostic and intraoperative applicability of this parameter.

## 1. Introduction

Ankle fractures are among the most common musculoskeletal injuries in adults and may be associated with concomitant disruption of the distal tibiofibular syndesmosis [1,2]. More complex ankle-region injuries, including intra-articular tibial pilon fractures, further underscore the importance of restoring osseous alignment, articular congruity, and stable distal tibiofibular relationships while carefully addressing associated soft-tissue injury. Syndesmotic injuries are particularly relevant in this context, as they have been reported in approximately 20% of ankle fractures [3,4,5]. Syndesmotic injuries of the ankle are complex and frequently underdiagnosed, leading to chronic instability and early-onset osteoarthritis if left untreated or inadequately reduced [6].

Conventional radiography remains the first-line imaging modality for the evaluation of distal tibiofibular syndesmotic injury, while computed tomography (CT), weight-bearing CT (WBCT), stress or comparative CT, and magnetic resonance imaging (MRI) provide complementary information according to whether osseous alignment, dynamic instability, or ligamentous integrity is being assessed [7,8,9,10,11]. On conventional radiographs, traditional parameters such as the tibiofibular clear space and tibiofibular overlap measured on anteroposterior and mortise views are widely used to assess syndesmotic alignment [1,3,6,12,13,14]. Although definitive assessment may require intraoperative stress testing or advanced imaging, fluoroscopic imaging with an image intensifier or direct radiography continues to play a central role in intraoperative evaluation [12,13]. However, these conventional measurements are highly dependent on rotational positioning and primarily reflect coronal-plane tibiofibular diastasis, thereby providing limited information on the multidirectional relationship of the distal tibiofibular syndesmosis.

Sagittal plane instability, specifically posterior translation of the fibula, is a subtle but critical component of syndesmotic injuries that is often overlooked on standard anteroposterior (AP) views. Although the lateral ankle radiograph is a standard component of the trauma series, it is primarily used to assess fracture patterns and talar subluxation, while standardized quantitative indices for evaluating the distal tibiofibular relationship remain limited, particularly for detecting small posterior translational malreductions [1,6,12]. Given these limitations of conventional radiography, CT-based assessment offers a more detailed evaluation of fibular translation, rotation, and asymmetry. However, reported measurements and reference values remain inconsistent, particularly for subtle sagittal malalignment. Building on previous work demonstrating the utility of three-dimensional modeling for controlled assessment of lower-extremity spatial relationships [15], this study used standardized CT-derived 3D models to investigate whether a simple lateral ratio-based measurement could quantify posterior fibular translation.

We hypothesized that standardized lateral-view ratio measurements would demonstrate predictable changes in response to controlled posterior translation of the fibula. Accordingly, this study aimed to develop and preliminarily assess candidate lateral tibiofibular ratios capable of distinguishing native syndesmotic anatomy from simulated 1 mm and 2 mm posterior fibular translation. The primary outcome was the receiver operating characteristic area under the curve (ROC AUC) for each candidate index, with particular emphasis on the proposed Lateral Tibiofibular Index (LTI), defined as (A + B)/(B + C). Given its 3D CT-based simulation design, this study was intended as an initial developmental investigation rather than a clinical diagnostic-accuracy validation study.

## 2. Materials and Methods

### 2.1. Study Design

The retrospective imaging dataset was obtained from the institutional lower-extremity CT angiography archive between 2017 and 2020. Institutional Review Board Approval for this study was granted by the Ankara City Hospital with the registration number E1-20-1309, and informed consent was waived because only previously acquired, anonymized imaging data were used. Adults aged 18 to 60 years were eligible.

**Figure 1:**
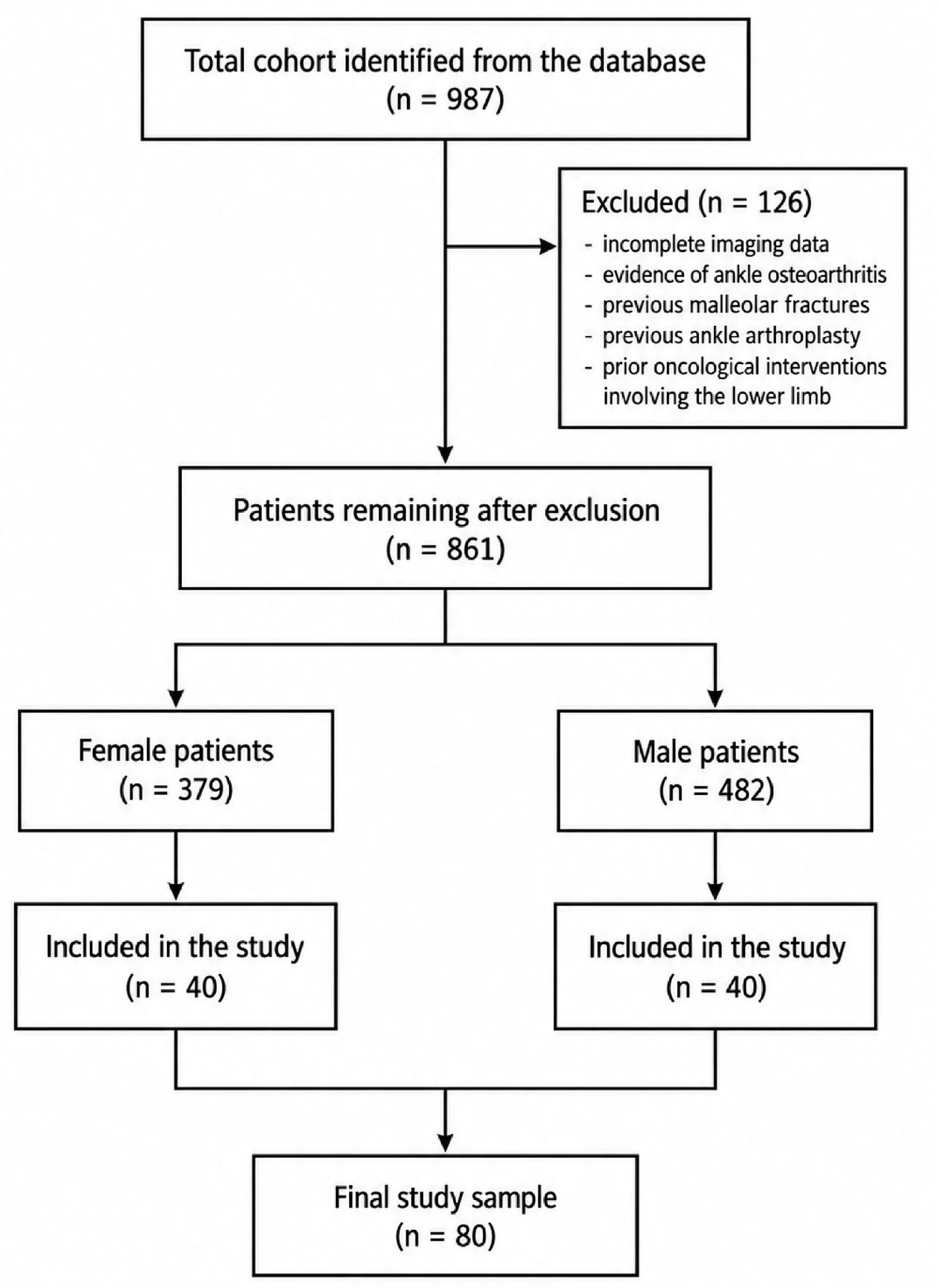
Flow diagram of participant selection.

Lower-extremity CT angiography examinations were selected because they provided high-resolution, thin-slice volumetric imaging of the distal tibia and fibula suitable for multiplanar reconstruction, three-dimensional segmentation, and controlled simulation of posterior fibular translation. The use of these datasets was therefore based on image quality and anatomical coverage rather than on the original vascular indication for scanning. Individual scan indications were not preserved in the retained de-identified research dataset. To minimize the potential influence of local pathological changes on tibiofibular morphology, patients with incomplete imaging data, ankle osteoarthritis, previous malleolar fractures, previous ankle arthroplasty, or prior oncological interventions involving the lower limb were excluded. The study cohort initially comprised 987 patients. Following the exclusion of 126 patients, 861 patients remained eligible for further assessment. The remaining cohort was stratified by sex, including 379 female and 482 male patients. After eligibility assessment, a sex-balanced sample of 80 participants (40 men and 40 women) was selected using random.org within the available eligible pool, yielding 160 bilateral ankles. A flow diagram illustrating the participant selection process is shown in Figure 1. Data were anonymized and processed using Mimics Innovation Suite (version 27.0; Materialise, Leuven, Belgium).

Because this study involved the development of a method based on an existing imaging archive—characterized as retrospective and exploratory—no prospective sample size calculation based on ROC analysis was performed. The final analysis sample consisted of 80 participants. Consequently, the precision of ROC estimates, threshold performance, and agreement statistics was determined using 95% confidence intervals, and participant-level resampling was employed where necessary to account for the clustering effect. All CT scans were performed using a Siemens SOMATOM Emotion scanner (Siemens Healthineers, Erlangen, Germany) with the following parameters: 110 kV, 90 mAs, and a slice thickness of 1.2 mm.

### 2.2. Three-Dimensional Reconstruction and Simulated Translation

Three-dimensional anatomical models of the tibia and fibula were reconstructed using semi-automated segmentation in Mimics Medical 27.0 (Materialise, Leuven, Belgium). Bone tissue was isolated using a standardized Hounsfield unit (HU) threshold of 200–2250 HU. The tibia and fibula were segmented as separate masks to allow for distinct morphometric analysis. Segmentation was performed by an investigator with extensive experience in Materialise-based 3D image processing. Automated thresholding provided satisfactory delineation of the tibia and fibula, and no manual correction was required. Primary measurements were performed by the investigator responsible for 3D image processing. For interobserver reliability analysis, a second investigator, blinded to the first observer’s measurements and to the condition labels, independently measured A, B, and C and calculated the LTI once for all native, 1 mm, and 2 mm configurations using the same predefined protocol.

The total length of the tibia was measured and divided into thirds. To establish the tibial anatomical axis, circumferential markers were placed at the junctions of the proximal and distal thirds of the tibial shaft [16]. Using the “Best-Fit to Surface” tool, spheres were generated at these landmarks, and the anatomical axis was defined by the line intersecting the center points of these spheres. An ankle plane, perpendicular to the tibial anatomical axis, was then reconstructed using the “Plane Normal to Curve” function. The measurement plane was centered at the intersection of the most superior articular surface of the ankle joint and the tibial axis and then positioned 10 mm proximal to the tibial plafond, consistent with commonly used radiographic and CT-based assessment levels for the distal tibiofibular syndesmosis [10,11]. Finally, the 3D models were exported to Materialise 3-matic (Materialise, Leuven, Belgium), where a high level of transparency was applied to simulate a lateral radiographic projection.

**Figure 2:**
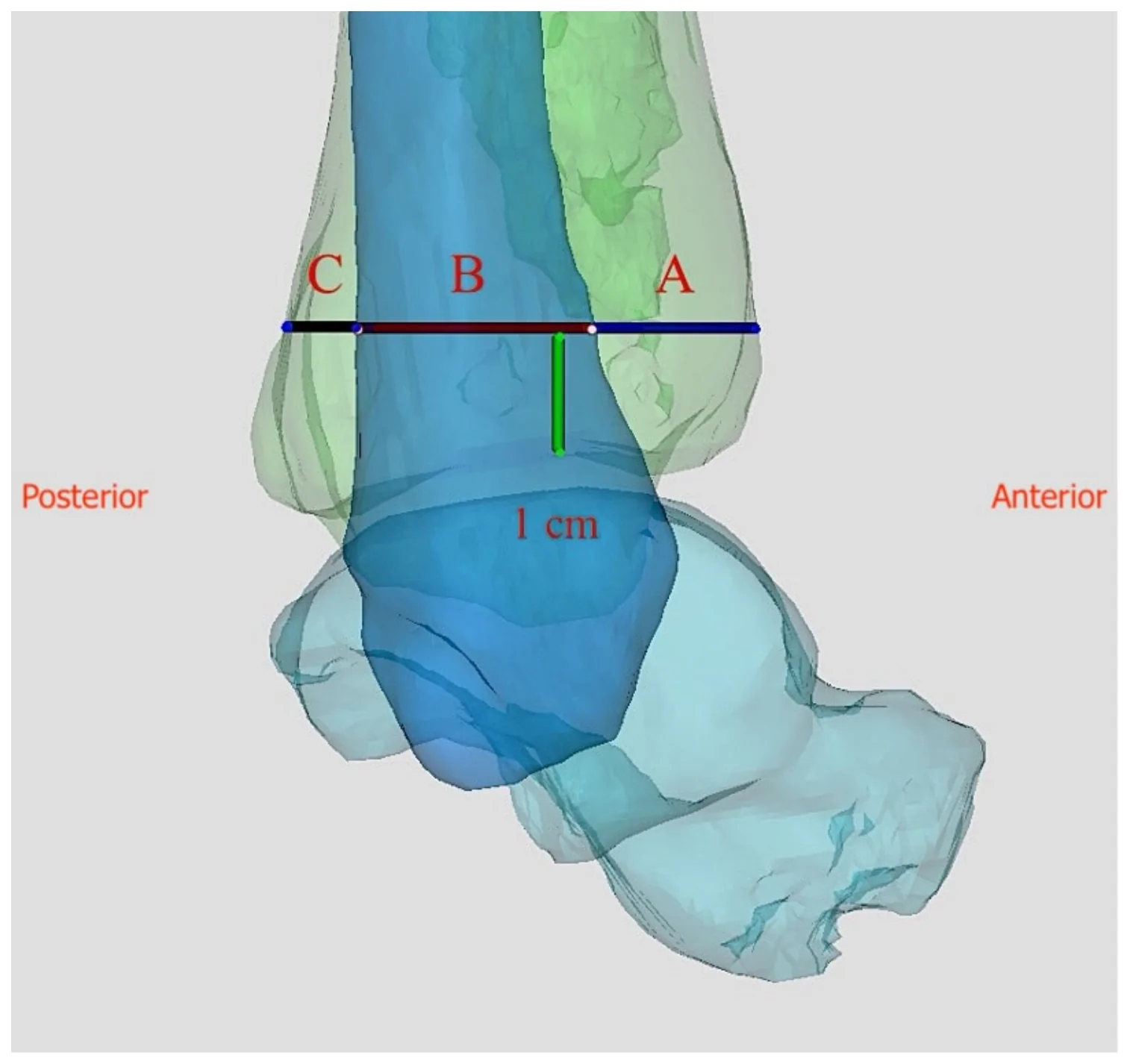
Definition of the lateral measurements in a standardized 3D CT-derived true-lateral projection obtained 10 mm proximal to the tibial plafond. A, distance between the anterior tibial cortex and anterior fibular cortex; B, fibular anteroposterior width; C, distance between the posterior fibular cortex and posterior tibial cortex.

Following the methodology of Koenig et al., the rotational position of the ankle was standardized to achieve a single-density view of the tibial plafond on the lateral view [1]. Three linear anatomical parameters (A, B, and C) were defined and measured at the level of the ankle plane on the lateral projection (Figure 2):

- Parameter A: The distance between the most anterior cortex of the tibia and the most anterior cortex of the fibula.
- Parameter B: The width of the fibula (distance between the anterior and posterior fibular cortex).
- Parameter C: The distance between the most posterior cortex of the fibula and the most posterior cortex of the tibia.

**Figure 3:**
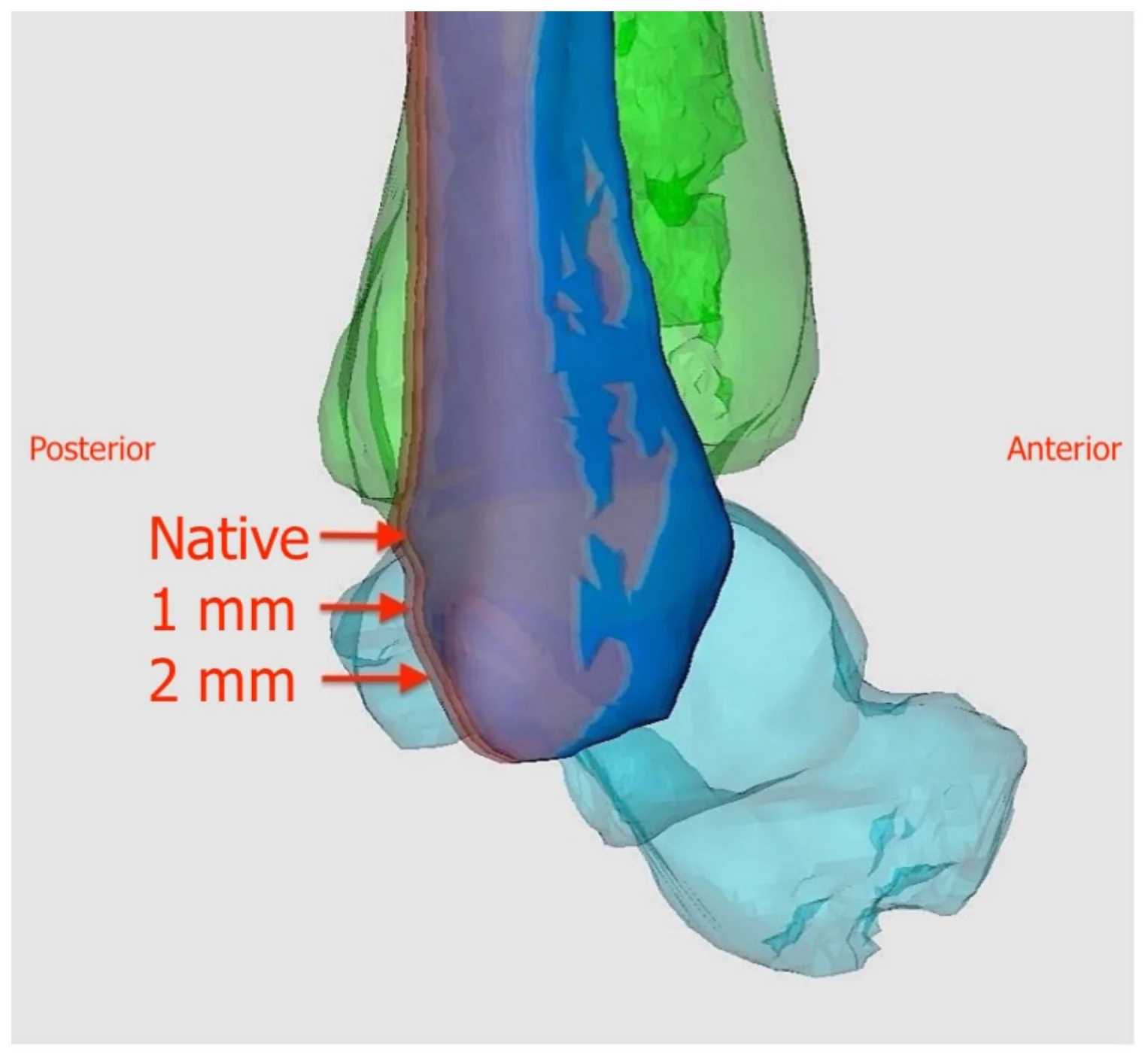
Controlled simulation of native alignment, 1 mm posterior fibular translation, and 2 mm posterior fibular translation. Translation was applied in a single posterior direction while the tibial position and projection geometry were kept constant.

These native parameters were recorded for all 160 ankles in the study group. Subsequently, a posterior fibular translation was simulated using the 3D anatomical models. Using the “Interactive Translate” function in the Mimics software, the fibula was translated posteriorly by 1 mm and 2 mm relative to the tibia (Figure 3). For the 2 mm displacement condition, the fibula was translated directly 2 mm posteriorly from its native position rather than incrementally from the 1 mm state. The parameters (A, B, and C) were re-measured at each increment of displacement. These increments were used to test small, graded changes in sagittal alignment; they were not intended to define clinical thresholds or to reproduce the full biomechanics of clinical syndesmotic injury. The simulation therefore represented isolated uniplanar posterior translation without induced fibular rotation, syndesmotic widening, ligament disruption, fracture morphology, loading, or soft-tissue effects.

### 2.3. Modeling

To evaluate simulation-based discriminatory performance, four ratio-based candidate indices were calculated from A, B, and C: A/C, (A + B)/C, A/(B + C), and (A + B)/(B + C). Ratios were used to reduce the influence of magnification and absolute bone dimensions. The final ratio, (A + B)/(B + C), was termed the Lateral Tibiofibular Index (LTI). Because height, weight, and body mass index were not available in the retained dataset, no claim of independence from body size was made.

Bilateral anatomical agreement and observer reliability were treated as distinct concepts. Right-left comparisons of native ankles were used to characterize bilateral anatomical agreement. Interobserver reliability was reported as well.

### 2.4. Data Analysis

Statistical analyses were performed using JASP version 0.95.4 (JASP, University of Amsterdam, The Netherlands), with statistical significance set at *p* < 0.05. Normality was assessed using the Shapiro–Wilk test. Interobserver reliability of measurements A, B, C, and LTI was evaluated using two-way mixed-effects, absolute-agreement intraclass correlation coefficients (ICCs) with 95% confidence intervals [17]. Because both ankles from each participant were included, statistical dependence between bilateral observations was accounted for by clustering at the participant level; in bootstrap analyses, participants rather than individual ankles were resampled, thereby preserving all within-participant observations within the same cluster.

Receiver operating characteristic (ROC) analyses were performed separately for native versus 1 mm and native versus 2 mm simulated posterior fibular translation for the candidate indices A/C, (A + B)/C, A/(B + C), and (A + B)/(B + C). AUCs with 95% confidence intervals were calculated, and candidate LTI thresholds were identified using the Youden index. AUCs were compared using paired participant-level bootstrap differences, with Holm adjustment for the three comparisons involving the LTI. For all pairwise comparisons, ΔAUC was defined as AUC_LTI_ − AUC_comparator_; therefore, positive values indicate greater discriminatory performance of the LTI. An LTI value of 1.50, derived from the upper limit of the central 95% reference interval of the native distribution (mean + 1.96 × SD), was evaluated separately as a provisional simulation-derived reference threshold rather than an optimized diagnostic cut-off. Sensitivity, specificity, 95% cluster-bootstrap confidence intervals, and positive and negative likelihood ratios were calculated for this threshold. To assess interobserver reliability, a second investigator, blinded to the first investigator’s measurements, independently performed all native, 1 mm, and 2 mm measurements once using the same predefined measurement protocol. Bilateral agreement was assessed using two-way absolute-agreement ICCs and Bland–Altman analysis. Sex differences in patient-level mean native LTI were evaluated using Welch’s t test, and age association was evaluated using Pearson correlation. Height, weight, and BMI were not analyzed because these data were unavailable.

## 3. Results

The 80 participants had a mean age of 42.7 ± 12.4 years (range, 18–60); 40 (50%) were men and 40 (50%) were women. Native right-left measurements showed good bilateral anatomical agreement rather than observer reliability (Table 1). For the LTI, the bilateral ICC was 0.770 (95% CI, 0.662–0.848). Bland–Altman analysis showed a mean right-left LTI difference of −0.0068 with 95% limits of agreement from −0.139 to 0.126. Corresponding right-left biases for A, B, and C were −0.30 mm, +0.36 mm, and −0.19 mm, respectively.

**Figure 4:**
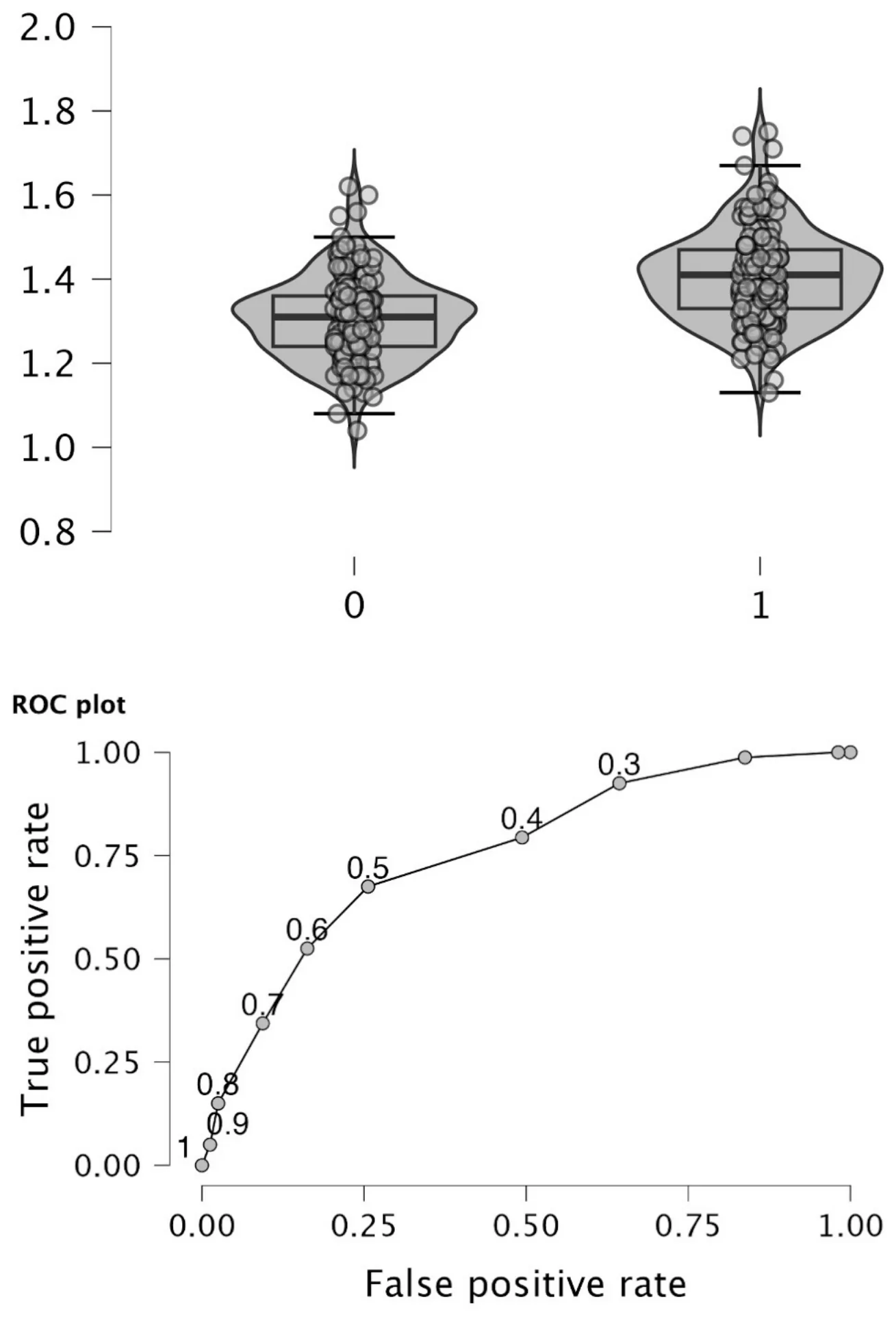
Distribution of the LTI in native and 1 mm simulated posterior translation configurations and the corresponding ROC curve. Native and translated observations originate from the same bilateral ankle models; inferential confidence intervals therefore account for participant-level clustering. The LTI AUC was 0.756 (95% CI, 0.727–0.793).

The LTI increased progressively across the simulated conditions: 1.306 ± 0.099 in native ankles, 1.406 ± 0.109 after 1 mm translation, and 1.515 ± 0.121 after 2 mm translation. For native versus 1 mm translation, the LTI AUC was 0.756 (95% cluster-bootstrap CI, 0.727–0.793), indicating moderate discrimination within the simulation (Table 2; Figure 4). The LTI had the numerically highest AUC; however, its AUC was not significantly greater than A/C (ΔAUC, 0.009; 95% CI, −0.008 to 0.026; Holm-adjusted *p* = 0.312).

**Figure 5:**
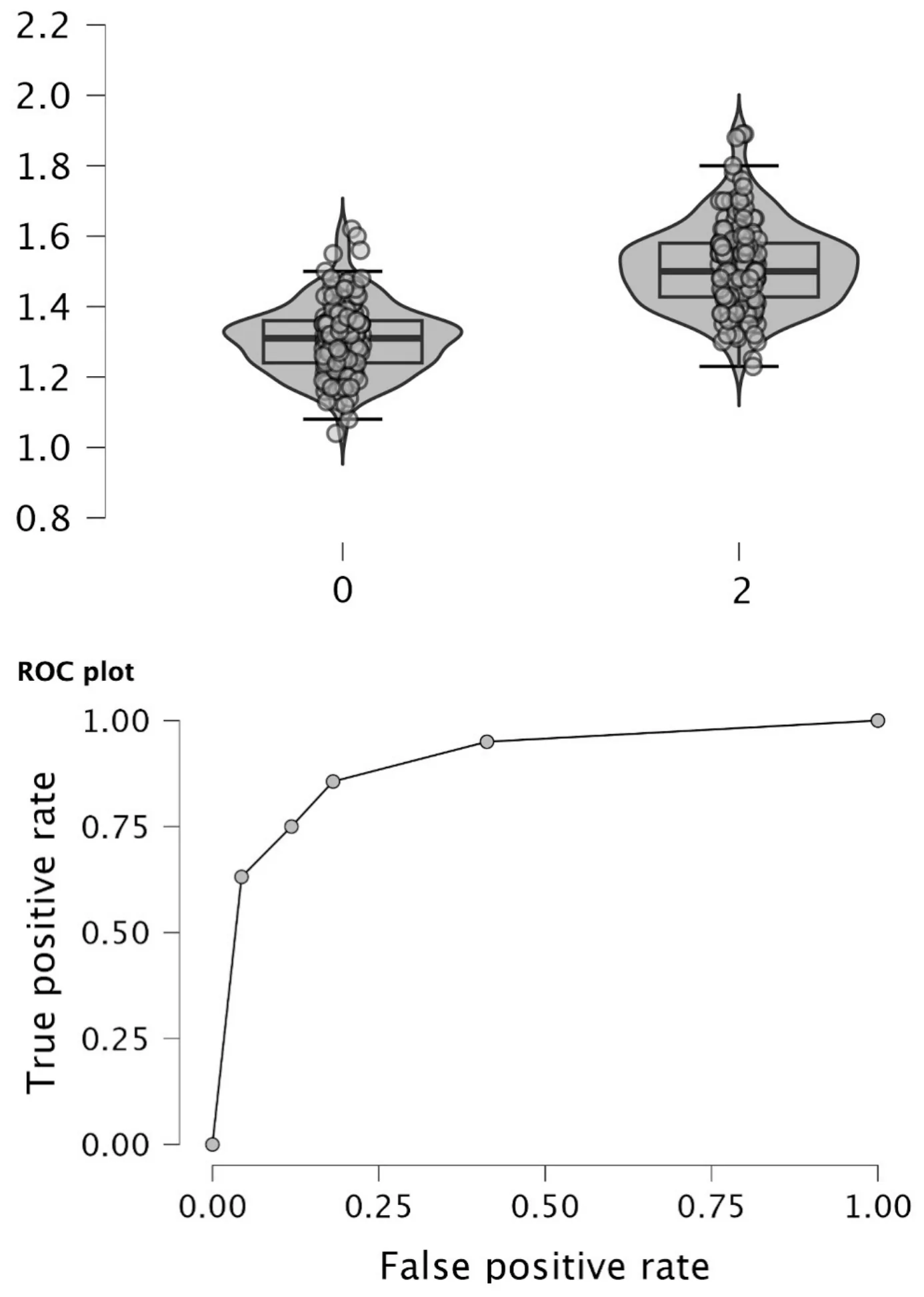
Distribution of the LTI in native and 2 mm simulated posterior translation configurations and the corresponding ROC curve. Native and translated observations originate from the same bilateral ankle models; inferential confidence intervals therefore account for participant-level clustering. The LTI AUC was 0.916 (95% CI, 0.885–0.946).

For native versus 2 mm translation, discrimination was substantially stronger. The LTI AUC was 0.916 (95% cluster-bootstrap CI, 0.885–0.946) (Table 3; Figure 5). Accordingly, the LTI showed the numerically highest AUC, but it was not significantly greater than A/C (ΔAUC, 0.008; 95% CI, −0.009 to 0.026; Holm-adjusted *p* = 0.348). The LTI did show significantly larger AUCs than (A + B)/C and A/(B + C) after Holm correction.

Interobserver ICCs for A, B, and C were in the good range across native, 1 mm, and 2 mm conditions (Table 4). These observer-reliability estimates are conceptually distinct from the bilateral right-left anatomical agreement reported in Table 1. Interobserver reliability was good across all simulated conditions, with LTI ICCs ranging from 0.85 to 0.89.

The native LTI distribution had a mean of 1.306 and an SD of 0.099, giving an upper reference limit of 1.501 using mean + 1.96 × SD. Thus, 1.50 was retained only as an approximate simulation-derived upper reference value. It was not the ROC-derived optimal discrimination threshold.

Youden analysis identified an LTI threshold of 1.357 for native versus 1 mm translation (sensitivity, 67.5% [95% CI, 58.1–76.3]; specificity, 74.4% [95% CI, 65.6–82.5]) and 1.381 for native versus 2 mm translation (sensitivity, 89.4% [95% CI, 82.5–95.0]; specificity, 80.6% [95% CI, 73.1–86.9]) (Table 5). In contrast, LTI ≥ 1.50 yielded sensitivity/specificity of 18.8% (95% CI, 11.9–25.6)/96.9% (95% CI, 93.1–99.4) for 1 mm translation and 55.6% (95% CI, 45.6–65.6)/96.9% (95% CI, 93.1–99.4) for 2 mm translation. Therefore, 1.50 was highly specific in this simulation dataset but insufficiently sensitive to serve as a general diagnostic threshold. Exploratory analyses showed no statistically significant difference in patient-level native LTI between men and women (mean difference, 0.020; 95% CI, −0.022 to 0.062; *p* = 0.343) and no significant correlation with age (*r* = 0.098; *p* = 0.388). Body-size effects could not be tested.

## 4. Discussion

The principal finding of this study is that the LTI changes systematically with controlled posterior fibular translation and shows stronger discrimination at 2 mm than at 1 mm in standardized 3D CT-derived lateral projections. The AUC was 0.756 for 1 mm translation, which should be interpreted as moderate simulation-based discrimination, and 0.916 for 2 mm translation, indicating substantially stronger separation. While the current literature has largely focused on defining normal anatomical relationships, our study defines the simulation-derived upper reference thresholds required to identify subtle sagittal plane instability, which is often overlooked in standard evaluations.

Accurate reduction in the syndesmosis is the most critical prognostic factor in ankle fractures [1,6,12]. However, assessing reduction, particularly in the sagittal plane, remains a significant challenge for orthopedic surgeons [1,12]. Koenig et al. demonstrated that even experienced trauma-trained surgeons have difficulty identifying posterior fibular displacement using standard fluoroscopy, with a sensitivity of only 68% for contralateral comparisons and a high failure rate in detecting 2.5 mm posterior displacements [1]. Similarly, Dikos et al. noted that while CT is superior for evaluating rotational and translational malreductions, it is not routinely available or practical in the intraoperative setting [13]. More recently, Abarca et al. reported that bilateral comparative fluoroscopy using a sagittal tibiofibular ratio could predict CT-confirmed malreduction in a prospective clinical cohort [18]. Our simulation-derived results suggest that utilizing quantitative ratios on lateral radiographs may help address this gap, demonstrating moderate discrimination for a simulated 1 mm posterior translation under standardized imaging conditions. In this study, the LTI is not a clinically validated diagnostic test; it is a quantitative index under development that demonstrates performance in distinguishing controlled posterior fibular translation on standardized 3D CT-derived lateral projections.

Ratio-based indices were selected to reduce dependence on absolute millimetric dimensions and magnification. Dikos et al. reported significant anatomical variations between genders in CT measurements, noting that men had significantly greater tibiofibular overlap and anterior tibiofibular intervals than women [13]. However, when these parameters were expressed as ratios of fibular width or incisura length, these gender differences disappeared. In the present sample, patient-level native LTI did not differ significantly by sex and was not significantly correlated with age. This finding is consistent with Yaradılmış et al., who analyzed the Turkish population and found that while AP radiographic measurements varied significantly by gender and age, the lateral Anterior Tibiofibular Ratio remained consistent across demographics [3]. However, height, weight, and BMI were unavailable; consequently, the present data do not support a claim that LTI is independent of body size or universally applicable across demographic groups. This point requires evaluation in larger, externally sampled clinical populations.

Several studies have described normal sagittal relationships of the distal tibiofibular joint. Croft et al. introduced the Anterior Tibiofibular Ratio, establishing that approximately 39% (±9%) of the tibia should be anterior to the anterior fibular cortex in an uninjured ankle [12]. Similarly, Grenier et al. described the Anteroposterior Tibiofibular ratio, reporting a mean value of 0.94 ± 0.13 in healthy subjects, suggesting that the anterior fibular cortex typically aligns with the center of the tibial physeal scar [6]. Yaradılmış et al. further validated the reliability of the Anterior Tibiofibular Ratio in a regional population, reporting a mean of 0.4 ± 0.1 [3]. Contemporary reviews emphasize that radiography, CT, MRI, stress/comparative CT, and WBCT answer different clinical questions [10,11]. WBCT and volumetric approaches are especially relevant to subtle instability because they permit three-dimensional evaluation under physiological loading, although thresholds and normal side-to-side variation remain active areas of investigation [7,9]. Accordingly, with further clinical evaluation, the LTI may provide a practical tool for addressing an important gap in the radiographic assessment of distal tibiofibular alignment.

However, these studies were limited to defining “native” anatomy and did not evaluate the diagnostic performance of these ratios in “pathological” situations of minimal displacement. Our study investigates this situation by simulating 1 mm and 2 mm posterior translations of the fibula. Among the candidate indices, the LTI showed the numerically highest AUC in both comparisons, with moderate discrimination for 1 mm translation (AUC, 0.756) and substantially stronger discrimination for 2 mm translation (AUC, 0.916). This contrasts with the visual assessment limitations highlighted by Koenig et al., providing a quantitative method for detecting malreduction [1]. Since both the threshold derivation and performance evaluation were performed on the same simulation dataset, the proposed threshold should be considered exploratory in nature and requires independent, prospective validation.

The purpose of this simulation-based study is to develop simple radiographic parameters that may ultimately facilitate intraoperative assessment of syndesmotic reduction and support decision-making without the need for advanced 2D or 3D imaging. Grenier et al. emphasized that lateral radiographic measurements are cost-effective and easily reproducible in the operating room [6]. Furthermore, due to the anatomical variations in the population, Koenig et al. and Dikos et al. both advocate for the use of the contralateral uninjured ankle as a reference standard [1,13]. Yaradılmış et al. also showed the high consistency of sagittal measurements between right and left ankles [3]. Future clinical studies should evaluate whether contralateral LTI measurement can serve as an individualized baseline for assessing syndesmotic reduction.

Threshold analysis showed that the proposed LTI value of 1.50 closely corresponded to the upper limit of the central 95% reference interval of the native LTI distribution (mean + 1.96 × SD). However, ROC-based optimization identified lower thresholds of 1.357 for 1 mm translation and 1.381 for 2 mm translation. Using an LTI threshold of 1.50 yielded high specificity (96.9%) but limited sensitivity, reaching 18.8% for 1 mm translation and 55.6% for 2 mm translation. Accordingly, an LTI value of 1.50 should be regarded as a provisional simulation-derived upper reference limit rather than an optimized diagnostic cut-off and may serve primarily as a hypothesis-generating value for future clinical studies.

Reliability is the cornerstone of any new radiographic measurement. Our analysis showed good intraclass correlation coefficients (0.81–0.89) for the measurements (A, B, and C), indicating that these parameters are consistent and reproducible. Unlike widely used AP-based parameters, which may vary substantially with tibial rotation, the lateral parameters defined in this study were measured on standardized true-lateral simulations, providing a more consistent basis for assessing distal tibiofibular alignment.

This study has some limitations. Because the study cohort was derived from patients who had undergone lower-extremity CT angiography, the sample may not fully represent an asymptomatic or population-based cohort. Although cases with local osseous or joint abnormalities that could directly affect distal tibiofibular morphology were excluded, the potential influence of unrecorded vascular or systemic comorbidities cannot be completely excluded. Accordingly, the absolute anatomical measurements should not be interpreted as population-based normative values. Additionally, this simulation study was based on artificial posterior translation of the fibula on 3D anatomical models. Although this approach allowed precise control of 1 mm and 2 mm displacement, it did not reproduce fibular rotation, syndesmotic widening, ligament disruption, fracture morphology, or soft-tissue constraints that may occur in true syndesmotic injuries. The models were also non-weight-bearing and generated under idealized true-lateral projection conditions, which may differ from routine radiographs and intraoperative fluoroscopy. Both ankles and repeated simulated states from the same participants were analyzed; although clustered resampling was used to account for this dependence, the sample included only 80 individuals. Therefore, future clinical studies comparing these indices on pre- and postoperative radiographs of patients with confirmed syndesmotic injuries are warranted before clinical application can be considered.

## 5. Conclusions

The LTI demonstrated promising discriminatory performance for distinguishing native ankle anatomy from controlled posterior fibular translation in standardized 3D CT-derived lateral projections, with substantially stronger performance at 2 mm than at 1 mm.

ROC analysis identified optimal thresholds of 1.357 for 1 mm translation and 1.381 for 2 mm translation. These findings support the LTI as a potentially useful quantitative parameter for detecting sagittal-plane fibular displacement under standardized imaging conditions.

These findings are preliminary and simulation-derived. Prospective studies involving patients with confirmed syndesmotic injury, clinically relevant reference standards, real radiographic or fluoroscopic acquisition, and independent validation cohorts are required before the LTI can be recommended for diagnosis, intraoperative decision-making, revision of reduction, or reduction in advanced imaging use.

## Figures and Tables

**Table 1:** Bilateral anatomical agreement between right and left native ankles.

	ICC	95% Confidence Interval
A	0.873	0.818–0.911
B	0.881	0.822–0.921
C	0.829	0.766–0.874
LTI	0.770	0.662–0.848

CI, confidence interval; ICC, intraclass correlation coefficient; LTI, Lateral Tibiofibular Index. A, distance between the anterior tibial cortex and anterior fibular cortex; B, fibular anteroposterior width; C, distance between the posterior fibular cortex and posterior tibial cortex. ICCs represent bilateral anatomical agreement between the right and left native ankles and should not be interpreted as interobserver measurement reliability.

**Table 2:** Clustered ROC analysis and formal AUC comparisons for native versus 1 mm simulated posterior fibular translation.

Index	AUC	95% CI	ΔAUC (LTI − Comparator)	95% CI for ΔAUC	Holm-Adjusted *p*
A/C	0.747	0.720–0.783	0.009	−0.008 to 0.026	0.312
(A + B)/C	0.718	0.693–0.750	0.038	0.011 to 0.064	0.023
A/(B + C)	0.731	0.702–0.767	0.025	0.001 to 0.049	0.090
LTI = (A + B)/(B + C)	0.756	0.727–0.793	Reference	-	-

AUC, area under the receiver operating characteristic curve; CI, confidence interval; LTI, Lateral Tibiofibular Index; ROC, receiver operating characteristic; ΔAUC, difference in AUC. ΔAUC was defined as AUC LTI—AUC comparator; therefore, positive values indicate a greater AUC for the LTI. Confidence intervals for ΔAUC are unadjusted participant-level bootstrap intervals, whereas *p* values are Holm-adjusted for the three pairwise comparisons with the LTI.

**Table 3:** Clustered ROC analysis and formal AUC comparisons for native versus 2 mm simulated posterior fibular translation.

Index	AUC	95% CI	ΔAUC (LTI − Comparator)	95% CI for ΔAUC	Holm-Adjusted *p*
A/C	0.908	0.878–0.937	0.008	−0.009 to 0.026	0.348
(A + B)/C	0.876	0.845–0.910	0.040	0.012 to 0.069	0.011
A/(B + C)	0.882	0.847–0.918	0.033	0.010 to 0.055	0.011
LTI = (A + B)/(B + C)	0.916	0.885–0.946	Reference	-	-

AUC, area under the receiver operating characteristic curve; CI, confidence interval; LTI, Lateral Tibiofibular Index; ROC, receiver operating characteristic; ΔAUC, difference in AUC. ΔAUC was defined as AUC LTI—AUC comparator; therefore, positive values indicate a greater AUC for the LTI. Confidence intervals for ΔAUC are unadjusted participant-level bootstrap intervals, whereas *p* values are Holm-adjusted for the three pairwise comparisons with the LTI.

**Table 4:** Interobserver reliability of A, B, C and LTI measurements in native, 1 mm, and 2 mm simulated posterior translation conditions.

Measurement Parameter	Native ICC	Native 95% CI	1 mm ICC	1 mm 95% CI	2 mm ICC	2 mm 95% CI
A	0.89	0.83–0.95	0.88	0.82–0.92	0.86	0.83–0.90
B	0.88	0.84–0.93	0.89	0.84–0.93	0.85	0.81–0.89
C	0.86	0.82–0.90	0.83	0.76–0.89	0.81	0.77–0.86
LTI	0.89	0.83–0.96	0.87	0.81–0.89	0.85	0.80–0.88

CI, confidence interval; ICC, intraclass correlation coefficient; LTI, Lateral Tibiofibular Index. A, distance between the anterior tibial cortex and anterior fibular cortex; B, fibular anteroposterior width; C, distance between the posterior fibular cortex and posterior tibial cortex. Interobserver reliability was assessed using a two-way mixed-effects, absolute-agreement, single-measurement ICC model. ICC values <0.50 were considered poor, 0.50–0.75 moderate, 0.75–0.90 good, and >0.90 excellent.

**Table 5:** LTI threshold performance in the simulation dataset. Confidence intervals were obtained by participant-level cluster-bootstrap resampling. The 1.50 value represents the native upper reference limit and was not selected by ROC optimization.

Comparison	Threshold Definition	LTI Cut-Off	Sensitivity (95% CI)	Specificity (95% CI)	LR+	LR−
Native vs. 1 mm	Youden optimal	1.357	67.5% (58.1–76.3)	74.4% (65.6–82.5)	2.63	0.44
Native vs. 1 mm	Upper reference value	1.500	18.8% (11.9–25.6)	96.9% (93.1–99.4)	6.00	0.84
Native vs. 2 mm	Youden optimal	1.381	89.4% (82.5–95.0)	80.6% (73.1–86.9)	4.61	0.13
Native vs. 2 mm	Upper reference value	1.500	55.6% (45.6–65.6)	96.9% (93.1–99.4)	17.80	0.46

CI, confidence interval; LTI, Lateral Tibiofibular Index; LR+, positive likelihood ratio; LR−, negative likelihood ratio; ROC, receiver operating characteristic. Confidence intervals for sensitivity and specificity were obtained using participant-level cluster-bootstrap resampling. The Youden-optimal threshold was defined as the value maximizing sensitivity + specificity − 1. The LTI value of 1.50 represents the simulation-derived upper reference limit of the native distribution and was evaluated separately from the ROC-derived optimal thresholds; it should not be interpreted as a clinically validated diagnostic cut-off.

## Data Availability

The raw data supporting the conclusions of this article will be made available by the authors on request.

## Abbreviations

The following abbreviations are used in this manuscript:

LTI	Lateral Tibiofibular Index
AP	Anteroposterior
3D CT	Three-Dimensional Computed Tomography
ICC	Intraclass Correlation Coefficient
ROC	Receiver Operating Characteristic
AUC	Area Under the Curve
CI	Confidence Interval
CT	Computed tomography
WBCT	Weight-Bearing Computed Tomography
SD	Standard Deviation
BMI	Body Mass Index
